# Effects of different flow patterns and end-inspiratory pause on oxygenation and ventilation in newborn piglets: an experimental study

**DOI:** 10.1186/1471-2253-14-96

**Published:** 2014-10-22

**Authors:** Carlos Ferrando, Marisa García, Andrea Gutierrez, Jose A Carbonell, Gerardo Aguilar, Marina Soro, Francisco J Belda

**Affiliations:** Anesthesiology and Critical Care Department, Hospital Clínico Universitario of Valencia, Av. Blasco Ibañez, 17, CP: 46010 Valencia, Spain

**Keywords:** Mechanical Ventilation, Pediatrics, Flow waveform, Oxygenation, Ventilation

## Abstract

**Background:**

Historically, the elective ventilatory flow pattern for neonates has been decelerating flow (DF). Decelerating flow waveform has been suggested to improve gas exchange in the neonate when compared with square flow (SF) waveform by improving the ventilation perfusion. However, the superiority of DF compared with SF has not yet been demonstrated during ventilation in small infants. The aim of this study was to compare SF vs. DF, with or without end-inspiratory pause (EIP), in terms of oxygenation and ventilation in an experimental model of newborn piglets.

**Methods:**

The lungs of 12 newborn Landrace/LargeWhite crossbred piglets were ventilated with SF, DF, SF-EIP and DF-EIP. Tidal volume (VT), inspiratory to expiratory ratio (I/E), respiratory rate (RR), and FiO_2_ were keep constant during the study. In order to assure an open lung during the study while preventing alveolar collapse, a positive end-expiratory pressure (PEEP) of 6 cmH_2_O was applied after a single recruitment maneuver. Gas exchange, lung mechanics and hemodynamics were measured.

**Results:**

The inspiratory flow waveform had no effect on arterial oxygenation pressure (PaO_2_) (276 vs. 278 mmHg, p = 0.77), alveolar dead space to alveolar tidal volume (VDalv/VTalv) (0.21 vs. 0.19 ml, p = 0.33), mean airway pressure (Pawm) (13.1 vs. 14.0 cmH_2_O, p = 0.69) and compliance (Crs) (3.5 vs. 3.5 ml cmH_2_O^−1^, p = 0.73) when comparing SF and DF. A short EIP (10%) did not produce changes in the results.

**Conclusion:**

The present study showed that there are no differences between SF, DF, SF-EIP and DF-EIP in oxygenation, ventilation, lung mechanics, or hemodynamics in this experimental model of newborn piglets with healthy lungs.

## Background

Historically, the elective intraoperative ventilatory flow in small infants has been decelerating flow (DF) [1, 2]. This was due to because the traditional thinking that a decelerating flow waveform (inherent to pressure control ventilation, PCV) improves oxygenation compared with the square flow waveform (common in volume control ventilation, VCV) related to a better intrapulmonary gas distribution. However, the superiority of decelerating flow compared with square flow has not yet been demonstrated during ventilation in small infants. Even in adults, the superiority has been questioned in clinical studies showing contradictory results [3–10].

Most studies [3–6] used VCV and PCV modes when comparing square vs. decelerating flow waveforms in terms of oxygenation and ventilation. However different VT between both modes could affect gas exchange. In order to prevent the changes in VT that can occur on PCV, we used VCV with decelerating flow as a surrogate of PCV because they show identical airway pressures and flow and volume waveforms under passive conditions when the same VT is administered.

There are no reported studies comparing square flow and decelerating flow in small infants with healthy lungs. Based on recent data, we hypothesized that in the non-atelectasic healthy lungs of small infants there are no differences in intrapulmonary gas distribution between squared and decelerating flow.

The primary outcome was to elucidate the differences in oxygenation between square and decelerating flow during ventilation in an experimental model of newborn piglets with healthy lungs at the same VT. A secondary outcome was a difference in ventilation and respiratory mechanics between the two flow waveforms.

## Methods

An experimental, prospective, controlled study was conducted. Approval was granted by the Ethical Committee for Experimental Research at the University of Valencia, Spain (Chairperson, Prof Dr. M. Real). Landrace/Large White crossbred female piglets weighing 2.9-3.1 kg and 7 days of age were used in the study. At the end of the experimental protocol, the animals were euthanized with an overdose of potassium chloride under deep anesthesia.

### Anesthesia management

Animals were premedicated with an intramuscular bolus of ketamine (1 mg Kg^−1^), medetomidine (0.06 mg Kg^−1^), and azaperone (0.06 mg Kg^−1^). Midazolam (1 mg Kg^−1^) and fentanyl (0.03 mg kg^−1^) were then administered in order to induce anesthesia. Endotracheal intubation was performed with a 3-mm internal diameter cuffed tube to prevent changes in VT due to air-leakage as clinically recommended [11]. During the study period, anesthesia was maintained with propofol (8 mg kg^−1^ min^−1^), remifentanil (0.15 μg kg^−1^ min^−1^), and cisatracurium (0.1 mg kg^−1^ h^−1^). Body temperature was maintained at 35- 36°C with a heat blanket.

### Instrumentation

A 3-Fr thermodilution catheter (PV2013L07-A, Pulsion Medical Systems AG, München, Germany) was inserted by a cut down to the right femoral artery for cardiac output monitoring. A 4-Fr double-lumen catheter (AK-14412, Arrow International, Inc, USA) was inserted by a cut down into the right or left internal jugular vein for drugs and fluid administration, and for transpulmonary thermodilution.

### Mechanical ventilation (MV)

**Figure 1:**
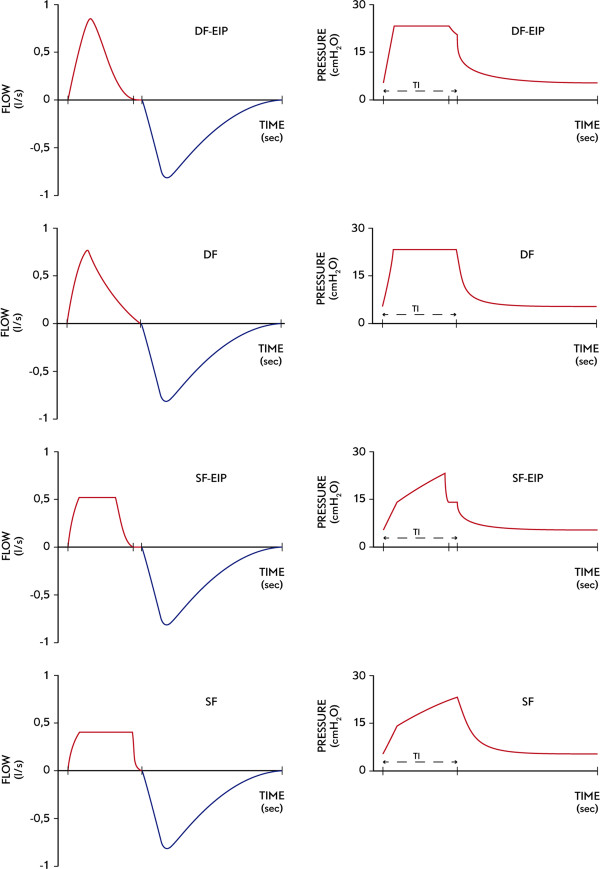
**Representative flow patterns studied with their respective pressure–time curve, all at the same tidal volumen, respiratory rate, positive end-expiratory pressure and inspiratory to expiratory ratio.** DF-EIP: decelerating flow with 10% end-inspiratory pressure, DF: decelerating flow, SF-EIP: square flow with 10% end-inspiratory pressure, SF: square flow. TI: Inspiratory time.

For mechanical ventilation, a Galileo gold (Hamilton, Bonaduz, Switzerland) was used in the pediatric mode. The ventilator allows square and decelerating flow, and end-inspiratory pause can be adjusted for a constant I/E relationship in VCV. The following ventilatory modes were compared in the study (Figure 1):

- ● Mode SF: Square flow, no end-inspiratory pause.
- ● Mode DF: Decelerating flow, no end-inspiratory pause.

Also, by adjusting a short end-inspiratory pause, another two modes were possible. The 10% end-inspiratory pause is based on our routine clinical practice, and due to a lack of evidence for the best short end-inspiratory pause duration in healthy lungs:

- ● Mode SF-EIP: Square flow with an end-inspiratory pause of 0.06 s (10% TI).
- ● Mode DF-EIP: Decelerating flow with an end-inspiratory pause of 0.06 s (10% Ti).

After induction of anesthesia, mechanical ventilation was initiated in volume-controlled ventilation (VCV) mode with a constant inspiratory flow (square wave) and the protective [12, 13] tidal volume (VT) set at 10 mL kg^−1^, inspiratory to expiratory ratio (I/E) at 1/2, respiratory rate (RR) of 30 breaths/min, and FiO_2_ of 0.5.

In order to assure a fully open lung during the study while preventing any alveolar collapse, a single recruitment maneuver (RM) was performed before starting the experimental ventilatory protocol. This consisted of the application of 40 cmH_2_O of continuous positive airway pressure (CPAP) for 10 seconds as described elsewhere [14], and adjusting for a PEEP of 6 cmH_2_O thereafter. Response to RM and adequacy of the PEEP was confirmed by checking for a normal alveolar-arterial oxygen gradient (279 ± 20 mmHg) in the baseline control of the first ventilatory mode [15, 16]. No further RMs were performed, and the PEEP-level was kept constant during the whole experiment. In order to prevent de-recruitment, the sequence of changes in ventilatory modes was performed without disconnecting the breathing circuit.

#### Respiratory monitoring

Volumetric capnography was recorded continuously using a NICO capnograph (Respironics, Wallingford, CT, USA) connected to a laptop running DataColl software (Respironics, Wallingford, CT, USA). The mainstream capnograph sensor (single-patient airway adapter, neonatal: 6312–00) was placed between the endotracheal tube and the “Y” piece of the breathing circuit. Expired volume and CO_2_ data were downloaded into a custom MatLab program (Mathworks, Natick, MA, USA) that constructed breath-by-breath volumetric capnograms for offline analysis.

The VTCO_2_,br is the amount of CO_2_ eliminated during one breath, which is obtained by integration of expired airway flow and PCO_2_. PEtCO_2_ is the partial pressure of CO_2_ at the end of expiration. Airway dead space (VDaw) was measured as the inflection point of phase II of the volumetric capnogram. Physiological dead space (VDphys) to tidal volume ratio (VD/VT) was calculated using the Bohr–Enghoff formula:


where PE´CO_2_ is the mixed PCO_2_ of an expiration. VDphys was then calculated by multiplying VD/VT and tidal volume. Alveolar dead space (VDalv) was obtained by subtracting VDaw from VDphys, which was then normalized by the alveolar tidal volume (VDalv/VTalv).

Peak inspiratory pressure (PIP) and mean airway pressure (Pawm) were determined through the NICO monitor with the pressure transducer placed between the endotracheal tube and the “Y” piece of the breathing circuit. Crs was automatically calculated as VT/(plateau pressure (Pplat) – PEEP).

Values of pH, PaO_2_, PaCO_2_, and bicarbonate were obtained from arterial blood gas analysis (i-STAT Analyzer, Abbott Laboratories, East Windsor, NJ, USA).

#### Hemodynamic monitoring and management

A PiCCO monitor (Pulsion Medical System AG, Munchen, Germany) was used for hemodynamic monitoring. Recent experimental studies using the PiCCO monitor in pigs have not reported any technical limitations [17, 18].

The cardiac index (CI) was obtained through transpulmonary thermodilution with the PiCCO monitor using the mean values of three 5-mL iced saline injections prior to each set of measurements. Mean arterial pressure (MAP) and heart rate (HR) were recorded continuously by arterial pulse wave analysis. All hemodynamic parameters were obtained with an atmospheric pressure calibration measured at the mid-thoracic level while the animals were in a supine position. Throughout the study, the animals received a continuous intravenous infusion of crystalloids (Lactate Ringer solution, 4–6 mL kg^−1^ h^−1^).

### Experimental sequence

To prevent bias while applying the four ventilatory modes (DF, SF, DF-EIP, SF-EIP) to the same animal, a sequence of all modes, starting and ending with the same mode, was designed (e.g., DF, SF, DF-EIP, SF-EIP, DF). The 12 possible combinations were determined, and the combinations were randomly applied to 12 animals.

After an initial hemodynamic stabilization, all animals were ventilated for 30 minutes with each mode. The VT, FiO_2_, PEEP, RR, and I/E relationships were kept constant during the study. To minimize error and variability, the mean value of the last 10 minutes of each mode was calculated for each variable of the ventilatory parameters. These values were considered representative of the effect of the mode (end-values) and were also taken as the baseline values for the following mode [19]. Mean values of the hemodynamic and arterial blood gas analysis (BGA) parameters were calculated from 4 measurements made during the last 10 min.

### Statistical analysis

Based on data from a previous study in healthy infants after cardiac surgery [7], it was estimated that a total of 12 animals would be needed to detect at least a 25-mmHg difference in oxygenation between flows, with a 5% significance level, and 80% power. All data were entered into the statistical package, SPSS version 15.0 (SPSS, Chicago, IL, USA). The Friedman test was performed for homogeneity. To compare primary outcomes, differences between the end-values and baseline measurements in each ventilatory mode, or the differences in the end-values between the four ventilatory modes, a statistical analysis using the Wilcoxon signed-rank test was performed [20]. To identify differences in the end-values between specific ventilatory modes (secondary outcome), the Bonferroni correction criteria was used to fit a type 1 risk to the chosen significance level (α = 0,05). All values are reported as the mean ± standard deviation (SD).

## Results

Table 1 shows mean ± SD of the oxygenation and ventilation parameters. No significant differences were found in any of the parameters measured when comparing end-values vs baseline in each ventilatory mode, with or without EIP (Table 2). No significant differences were found when the end-values of the four ventilatory modes were compared (Table 2). Table 2 shows the mean ± SD of the respiratory mechanic parameters. No significant differences were found in PIP, Pawm, and Crs when the end-values of the four ventilatory modes were compared. The mean end-values of CI and MAP were not significantly different between the four ventilatory modes (Table 3).

**Table 1:** **Oxygenation and ventilation parameters**

	MODE	Baseline	End	Difference between delta values		Difference between delta values of all modes	Difference between end-values of SF and DF	Difference between end-values of SF and SF _EIP_	Difference between end-values of DF and DF _EIP_
		Mean ± SD	Mean ± SD	Mean ± SD	p-value	p-value	p-value	p-value	p-value
**PaO**_**2**_	**SF**_**EIP**_	274 ± 22	283 ± 28	8 ± 22	0,58	0,19	0,77	0,25	0,17
	**DF**_**EIP**_	275 ± 26	279 ± 23	5 ± 21	0,75				
	**SF**	269 ± 25	276 ± 24	7 ± 18	0,36				
	**DF**	281 ± 22	278 ± 24	−4 ± 17	0,27				
**PaCO**_**2**_	**SF**_**EIP**_	39 ± 4	38 ± 5	−1 ± 4	0,54	0,76	0,42	0,16	0,53
	**DF**_**EIP**_	40 ± 7	39 ± 7	−1 ± 5	0,62				
	**SF**	39 ± 6	42 ± 6	1 ± 5	0,27				
	**DF**	39 ± 6	39 ± 5	0 ± 3	0,74				
**VDalv/VTalv**	**SF**_**EIP**_	0,20 ± 0,1	0,21 ± 0,1	0,01 ± 0,05	0,46	0,16	0,33	0,59	0,73
	**DF**_**EIP**_	0,22 ± 0,1	0,20 ± 0,1	−0,05 ± 0,03	0,51				
	**SF**	0,20 ± 0,1	0,19 ± 0,1	−0,01 ± 0,04	0,12				
	**DF**	0,20 ± 0,1	0,21 ± 0,1	0,04 ± 0,02	0,09				

Data are presented as mean ± SD. Delta: end – baseline values. SF: square flow, DF: decelerating flow, SF_EIP_: square flow with end-inspiratory pause, DF_EIP_: decelerating flow with end-inspiratory pause. PaO_2_: arterial oxygen tension (mmHg), PaCO_2_: arterial carbon dioxide tension (mmHg), VD/VT: alveolar dead space to alveolar VT ratio.

*when significant, P <0,05.

**Table 2:** **Respiratory mechanics parameters**

	SF	DF	SF _EIP_	DF _EIP_	Difference between end- values of all modes	Difference between end-values of SF and DF	Difference between end-values of SF and SF _EIP_	Difference between end-values of DF and DF _EIP_
					p-value	p-value	p-value	p-value
**Pawm**	13 ± 2	14 ± 1	13 ± 1	13 ± 2	0,56	0,69	0,72	0,22
**PIP**	23 ± 7	22 ± 2	21 ± 2	21 ± 1	0,51	0,68	0,47	0,44
**Crs**	3,5 ± 0,4	3,5 ± 0,5	3,5 ± 0,6	3,6 ± 0,2	0,39	0,73	0,87	0,36

Data are presented as mean ± SD. Delta: end – baseline values. SF: square flow, DF: decelerating flow, SF_EIP_: square flow with end-inspiratory pause, DF_EIP_: decelerating flow with end-inspiratory pause. Pawm: mean airway pressure (cm H_2_O) PIP: Peak inspiratory pressure (cm H_2_O), Crs: respiratory system compliance (ml cm H_2_O^−1^).

*when significant, P <0,05.

**Table 3:** **Hemodynamic parameters**

	SF	DF	SF _EIP_	DF _EIP_	Difference between end values in all modes
					p-value
**CI**	5,31 ± 1,6	5,15 ± 0,9	5,23 ± 1,2	5,59 ± 1,7	0,36
**MAP**	75 ± 12	83 ± 12	82 ± 16	81 ± 11	0,89

Data are presented as mean ± SD. CI: cardiac index (ml Kg^−1^ min^−1^), MAP: mean arterial pressure (mm Hg). *when significant, P <0,05.

## Discussion

This study shows no differences in oxygenation (PaO_2_), ventilation (VDalv/VTalv, PaCO_2_), lung mechanics (Crs), or hemodynamics (CI) between the square and the decelerating inspiratory flow waveform in this experimental setting of newborn piglets with healthy lungs. The same results were obtained when a short end-inspiratory pause of 10% was added to the inspiratory time during both square and decelerating flow.

To the best of our knowledge, until now the optimal flow pattern and the effects of an end-inspiratory pause in terms of oxygenation and ventilation during ventilation in an animal model of small lungs has not been thoroughly investigated.

Some studies [3–7, 9, 10, 21–26] compared square (common in VCV) vs. decelerating flow (inherent to PCV) in terms of gas exchange, lung mechanics, and hemodynamics. Most of these studies, clinical and experimental, were performed in adults with heterogeneous lungs (with acute lung injury, ALI), applied different methodologies, used different ventilator modes to compare flows (PCV for decelerating flow and VCV for square flow), and produced contradictory results.

Our results in an experimental model of small healthy lungs are similar to those obtained in other settings in healthy lungs. Kocis [7] found no differences in PaO_2_, PaCO_2_, MAP, and cardiac output (CO) between square and decelerating flow during postoperative cardiac surgery in infants with healthy lungs weighing over 5.5 kg when a level of 2–3 cmH_2_O of PEEP was set in all patients. It is true that our initial PaO2 was much higher than the PaO2 in the infants included in Kocis study. The reason may be justified by methodological differences. Our initial PaO2 was after a recruitment maneuver with a PEEP level that prevents alveolar collapse, and the initial PaO2 in the Kocis study was after cardiac surgery where there was probably a lung collapse, no recruitment maneuvers, and lower PEEP levels. Smith [8] compared 4 flow patterns and found no significant differences in PaO_2_, PaCO_2_ and VD/VT, MAP, and CO.

Contrary to our results, some studies found differences between square and decelerating flow when applied in adults with lung injury. Al-Saady et al. [3] and Davis et al. [4] compared square vs. decelerating flow and showed that decelerating flow improved PaO_2_, VD/VT, and Crs. The authors justified these results because the higher mean airway pressure (Pawm) produced by the decelerating flow favored alveolar recruitment and gas redistribution and diffusion. Higher Pawm generates more alveolar recruitment, which improves the ventilation/perfusion relationship. This effect is based on a mathematical model [25] and could be especially important in lungs with a high alveolar time constant [3] accounting for better gas exchange. However, this effect is not apparent in a homogeneous lung [26].

During mechanical ventilation, the infant respiratory system is prone to alveolar collapse [27–29], leading to a heterogeneous lung. In our study, in order to assure an homogeneous open lung (confirmed by a normal alveolar-arterial oxygen gradient), while preventing alveolar re-collapse, an alveolar recruitment maneuver (ARM) [14] was performed in all animals and 6 cmH_2_O of PEEP was applied afterwards (based on previous experimental studies) [30–32]. In this situation, the possible benefits of alveolar recruitment secondary to higher Pawm with decelerating flow would disappear [9, 10]. However, the ARM and the supraphysiological PEEP levels used in this study for keeping the lung open are not common in clinical practice because of the supposed increased risk of barotrauma in small infants due to over-distension. Recently, however, it was shown that higher airway pressures than those used to recruit healthy lung are needed to produce barotrauma in small lungs without chest wall [27].

The results of previous studies [9, 10, 23] confirm our hypothesis. Markström [10] showed no differences between square vs. decelerating flow in 13 pigs with ALI in PaO_2_, functional residual capacity (FRC) and Crs despite the differences observed in Pawm. As in our study, they performed an ARM at the start of the study and they compared both flows with different PEEP levels. However, the Markström study showed that PaCO_2_ was significantly lower with PCV, demonstrating better alveolar ventilation. Recent studies in adults using imaging techniques confirm our results. No differences in intrapulmonary gas distribution with computed tomography-scanning was found between square and decelerating flow [9, 23].

In relation to alveolar ventilation and dead space, some studies in injured patients found a lower PaCO_2_ and VD/VT with decelerating flow [3, 4, 9, 10, 24]. These results in lungs with different alveolar time constants could be justified by the theory of the mean distribution time (MDT) [33], which establishes that CO_2_ elimination is enhanced when the time available for gas distribution and diffusion within the respiratory zone increases (as occurs with decelerating flow because of higher initial peak flow). However, recently end-inspiratory flow (EIF) was shown to be a determinant of CO_2_ elimination. A high EIF enhances CO_2_ elimination, but an EIF of 0, as occurs with decelerating flow, worsens CO_2_ elimination and could balance the positive effects of decelerating flow in MDT [34]. This could be especially important in healthy lungs [34] and may explain our findings and those obtained by Smith et al. [8].

In relation to respiratory mechanics, several studies described higher PIP and lower Pawm with SF compared with DF. Differences could be significant in injured lungs with low compliance where PIP is lower with DF than with SF. As the flow decreases, the resistive pressure decreases, but the elastic pressure increases as the lungs fill [3]. However, several studies have not found such differences in situations of normal or high compliance [7, 9]. In contrast, in patients with high resistance as in our study because the use of small-sized endotracheal tubes, the pressures are initially highest using DF with the fastest flow and could remain elevated throughout the respiratory cycle [35].

### Effects of the end inspiratory pause (EIP)

Until now, the effects of end-inspiratory pause (EIP) on the V/Q relationship during mechanical ventilation of small infant were unknown. In order to ascertain if the effect of flow waveform on oxygenation and ventilation was influenced by EIP but predominantly prevented the effects of EIP at the same time, a short (10% of the Ti) was added in both flows without modifications in the VT and the I/E relationship. We show that a 10% EIP did not produce differences in oxygenation (PaO_2_) and ventilation (PaCO_2_, VDalv/VTalv) in this experimental model of newborn piglets. The results in oxygenation are in concordance with previous studies [36, 37] and are justified because EIP does not decrease shunt as it does not recruit alveoli. Different from previous studies (in healthy and injured lungs) [33, 34, 36–38], our results did not show differences in ventilation. The EIP could improve ventilation because [1] it favors gas redistribution in the lungs with different alveolar time constants, improving the V/Q relationship and therefore improving VD/VT [39]. This effect may not appear in homogeneous lungs without different alveolar time constants as in our model of normal lungs; and (2) the EIP increases the MDT. We did not observe an improvement in ventilation (PaCO_2_) with constant inspiratory time. When I/E relationship is constant, previous studies in healthy lungs [32, 40] showed that higher EIP (20%) is required to improve ventilation, especially with high RR.

As we observed in our results, hemodynamics remained constant throughout the experimentation with no clinical differences between flows. These results are consistent with previous studies [4, 7, 9, 24]. Therefore, the effects of different flow patterns in oxygenation and ventilation are not explained by changes in pulmonary perfusion.

Historically, the elective intraoperative ventilatory mode in very small infants has been pressure control ventilation for two basic reasons. First, the very real limitation of older anesthesia machines to guarantee a constant VT during volume control ventilation, and second, because of the traditional thinking that DF (inherent to pressure control) improves oxygenation compared with SF (common in volume control). The results obtained in our study together with the new anesthesia machines that accurately ensure very low VT [1] could make volume control the elective intraoperative mode.

Several limitations of this study need to be mentioned. Firstly, this is an experimental study which may limit the application of the findings to the clinical setting. Secondly, this study examined a small number of animals and therefore was not powered to expose small differences in some of the variables measured. Thirdly, currently there is no general indication for routinely applying ARM and supraclinical levels of PEEP during intraoperative ventilatory management in infants. By not applying these techniques, lung homogeneity is not guaranteed, and decelerating flow may favor redistribution of gas and a better V/Q relationship. Fourth, The crossover methodology used could mask differences in the end values of the modes if the effects of the previous ventilatory mode spilled over into the next ventilatory mode. Fifth, a possible limitation were the effects of an unblinded study, however biases was minimized with a standarized protocol in management, monitoring, measurements and data colection. Finally, the clinical monitors used in the study are not completely validated for use in newborn piglets; however, they were applied throughout the study, and their inherent percent error was considered to be similar for the four conditions assessed.

## Conclusions

In conclusion, the present study showed that there are no differences between square and decelerating flow, with or without EIP, in oxygenation, ventilation, lung mechanics, or hemodynamics in this experimental setting in an animal model of newborn piglets with healthy lungs. However, further studies are needed to elucidate whether or not different flow-waveforms may have a direct effect when ventilating small lungs with acute lung injury.

## Abbreviations

VDaw: Airway dead space
VDalv/VTalv: Alveolar dead space to alveolar tidal volume
PaO_2_: Arterial oxygenation pressure
VTCO_2_: br: Amount of CO_2_ eliminated during one breath
ALI: Acute lung injury
ARM: Alveolar recruitment maneuver
CI: Cardiac index
CO: Cardiac output
Crs: Compliance
CPAP: Continuous positive airway pressure
DF: Decelerating flow
EIF: End-inspiratory flow
EIP: End-inspiratory pause
FRC: Functional residual capacity
HR: Heart rate
I/E: Inspiratory to expiratory ratio
MAP: Mean arterial pressure
MDT: Mean distribution time
Pawm: Mean airway pressure
MV: Mechanical Ventilation
PE´CO_2_: Mixed PCO_2_ of an expiration
PEtCO_2_: Partial pressure of CO_2_ at the end of expiration
VDphys: Physiological dead space
PIP: Peak inspiratory pressure
Pplat: Plateau pressure
PEEP: Positive end-expiratory pressure
PCV: Pressure control ventilation
RR: Respiratory rate
RM: Recruitment maneuver
SD: Standar deviation
SF: Squared flow
VT: Tidal volume
VCV: Volume control ventilation.
